# Micro/nano hierarchical structured titanium treated by NH_4_OH/H_2_O_2_ for enhancing cell response

**DOI:** 10.1371/journal.pone.0196366

**Published:** 2018-05-03

**Authors:** Xin Yuan, Yi Kang, Jun Zuo, Youneng Xie, Li Ma, Xuelei Ren, Zeyu Bian, Qiuping Wei, Kechao Zhou, Xiyang Wang, Zhiming Yu

**Affiliations:** 1 State Key Laboratory of Powder Metallurgy, School of Materials Science and Engineering, Central South University, Changsha, PR China; 2 The Third Xiangya Hospital, Central South University, Changsha, PR China; 3 Xiangya Stomatological Hospital, Central South University, Changsha, PR China; 4 Hunan Provincial Engineering Laboratory for High-performance Bio-engineered Biomimetic Bone Materials, Xiangya Hospital, Central South University, Changsha, PR China; Institute of Materials Science, GERMANY

## Abstract

In this paper, two kinds of titanium surfaces with novel micro/nano hierarchical structures, namely Etched (E) surface and Sandblast and etched (SE) surface, were successfully fabricated by NH_4_OH and H_2_O_2_ mixture. And their cellular responses of MG63 were investigated compared with Sandblast and acid-etching (SLA) surface. Scanning electron microscope (SEM), Surface profiler, X-ray photoelectron spectroscopy (XPS), and Contact angle instrument were employed to assess the surface morphologies, roughness, chemistry and wettability respectively. Hierarchical structures with micro holes of 10–30 μm in diameter and nano pits of tens of nanometers in diameter formed on both E and SE surfaces. The size of micro holes is very close to osteoblast cell, which makes them wonderful beds for osteoblast. Moreover, these two kinds of surfaces possess similar roughness and superior hydrophilicity to SLA. Reactive oxygen species were detected on E and SE surface, and thus considerable antimicrobial performance and well fixation can be speculated on them. The cell experiments also demonstrated a boost in cell attachment, and that proliferation and osteogenic differentiation were achieved on them, especially on SE surface. The results indicate that the treatment of pure titanium with H_2_O_2_/NH_4_OH is an effective technique to improve the initial stability of implants and enhance the osseointegration, which may be a promising surface treatment to titanium implant.

## Introduction

Surface modification is wildly utilized to decorate the titanium implants in order to obtain specific physical morphologies and chemical composition, forming a biological binding between the implant and surrounding bone and thus improving their biocompatibility. It has been attracting an increasing attention in the field of implanted materials [1–4]. Sandblast and acid-etching (SLA) is one of the most prevalent processing technologies for titanium implants. However, the surface obtained by this method always exhibit obvious poor hydrophilicity, which directly affect the interactions among biological fluids, tissues, cells and implant [5–7]. More importantly, SLA surfaces with micron/submicron rough structures can be immediately loaded under certain conditions. Meanwhile, in vitro experiments also found that SLA surface can effectively promote osteogenic differentiation, while the adhesion and proliferation abilities of cells as well as the healing process still remain to be improved due to the lack of nanostructures [3, 8]. Titanium implants with nano-structured surfaces have higher surface energy and better wettability, which can absorb more fibrin and matrix protein and promote growth and transformation factors. Therefore, osteoblast adhesion will be increased, and the process of bone integration also will be accelerated [9–11]. However, the nanostructures of the implant cannot completely replace the microstructures. It has been found that the single nanoscale morphology is not enough to ensure a solid osseointegration [12, 13]. The combination of micro and nano structure shows a synergistic effect, promoting bone cell differentiation and improving the implant-bone binding [14, 15]. The micro-scale surface of the implant is favorable for the formation of mechanical locking between the implant surface and the surrounding tissue, which is conducive to the formation of superior initial stability [16]. However, the nano-scale surface has a significant advantage in inducing cell proliferation, differentiation and favorable bone formation [17]. A fine nano-hole structure was obtained on the surface of pure titanium by using the mixture of sulfuric acid and hydrogen peroxide, but there are no other structures at a micron level [18–21]. Similarly, the pure titanium treated with concentrated sodium hydroxide solution at a high temperature shows a nano-network structure, but the surface in the micron level is almost smooth [22, 23]. SLA, as a relatively mature process, can be used to get good micron roughened surfaces without obvious material defects, but problems such as the lacking nano-structure as well as poor hydrophilicity cannot be ignored.

H_2_O_2_ is an excellent oxidant to titanium with clean oxidation products and sensible reactions. And O_2_^2−^, a hydrophilic group, could still exist on the etched implant surface after being etched by H_2_O_2_, which can also inhibit bacterial reproduction. Xie et al. [24] fabricated a novel surface combining SLA micron morphology with nano-structures by using H_2_O_2_, and the surface hydrophilicity was significantly improved. Further, the micron/sub-micron rough surfaces were obtained by double etched process that firstly TA2 titanium plates were etched in a mixture of aqueous ammonia and hydrogen peroxide and subsequently etched in a mixture of hydrochloric acid and sulfuric acid. Reactive oxygen species (ROS) were detected on the surface, and the hydrophilicity of the material was also enhanced [25]. Studies have also indicated that the presence of ROS can prohibit bacterial breeding and accelerate wound healing and osseointegration [26–29]. Therefore, a mixture of ammonia and hydrogen peroxide may be a good candidate for obtaining clean surfaces with micro-nano composite structures.

Generally, Sandblast acid-etched (SLA) is the most commonly used technique in surface treatment of titanium implant at present. However, the treated surface by SLA just has micro and sub-micro structure and poor wettability, leading the poor cell attachment, proliferation and differentiation. In order to solve these problems, we etched titanium samples by one-step etching with NH_4_OH and H_2_O_2_ instead of SLA to obtain a hierarchical structure with ROS on it. Micro/nano hierarchical structures can be obtained by one-step etching with NH_4_OH and H_2_O_2_, and the pretreatment process of sandblasting is also added to further roughen the surface. The results show that the modified surface of SE has micro/nano hierarchical structures and better wettability because of the existence of Reactive Oxygen Species (ROS). The SE samples promote cell proliferation and differentiation compared with SLA. The results of SEM indicate that it also obtains micro/nano hierarchical structures which is similar to SE. Though the cell results are inferior to SE, it is also better than SLA. In this study, we focus on the effects of hierarchical structures on surface properties and cell responses after modified by a mixture of H_2_O_2_ and NH_4_OH. With SLA surfaces as a control group, surface properties and cell responses were systematically investigated, aiming at providing a possible way for the processing technology of pure titanium implants.

## Materials and methods

### Sample preparation

TA4 commercial pure titanium plates (Φ15 mm) were mechanically polished and ultrasonically cleaned in acetone, ethanol and deionized water for 10 minutes, respectively. The clean polished samples were then etched in mixed H_2_O, HNO_3_ and HF (85:35:5, v/v) solution for 40 s and then sonicated with deionized water for 2 min. After drying, the samples were ultrasonically etched in a mixture of hydrogen peroxide and aqueous ammonia for 2 h at a temperature of 45°C. Then the samples were ultrasonically cleaned with deionized water for 15 minutes, which named as Etched (E) group. Polished titanium plates were sandblasted by Al_2_O_3_ (Φ250~500 μm) at a pressure of 3 atm until the surfaces were uniformly gray. The sandblasted samples were also treated like polished samples above at the same way, which named as Sandblast and etched (SE) group. The blasted titanium plates were etched in boiling mixed H_2_SO_4_ and HCl solution to obtain the Sandblast and acid etching(SLA) surfaces.

### Surface characterization

The surface topography has a large effect on the performance of pure titanium implants. Scanning electron microscopy (SEM, Nova NanoSEM 230) was employed to observe the surface morphology. The surface roughness of the sample was measured by a surface profiler (Dektak 150 surface profiler). The surface chemical state was determined by X-ray photoelectron spectroscopy (XPS, ESCALAB 250Xi). The surface wettability property of the materials was evaluated by contact angle instrument, pure water was used as a test reagent and contact angle was tested by sessile drop method.

### Cell culture

Three groups of titanium plates were disinfected and placed in 24-well plates, and then MG63 (Shanghai Institutes for Biological Sciences, China) cells were seeded on the surface of titanium plate. The culture conditions for MG63 cells is DMEM high glucose, 10% FBS, 1% double antibody. The 24-well plates were incubated in a 5% CO_2_, 37°C and 100% humidity incubator. Three replicates were set up.

### Cell adhesion

After 4 hours of cultivation, the cells were rinsed with buffer solution (PBS) to wash out non-adherent cells, and then fixed with 4% paraformaldehyde (Sigma, USA) for 15 minutes. After staining with AO solution for 10 minutes, cells were fluorescence measured. When observing the cell adhesion morphology, fluorescence microscope photographs were taken after fixing them 15min in 4% paraformaldehyde and incubating 1h in Rhodamine phalloidin. The area of cell adhesion was calculated by ImageJ software.

### Cell proliferation and viability

Cell counts were measured with automatic counting device after culturing them 24 h, 48 h, 72 h respectively at the condition mentioned above. For CCK8 assay, 10% CCK8 solution was placed in complete medium and 300 μl per well for 24 well plates. After incubation for 4 hours, the supernatant was aspirated into a 96-well plate, 100ul per well, and 450nm absorbance was measured using a microplate reader. The absorbance of 2×10^4^ cells was the initial value.

### Cell differentiation

Three groups of titanium plates were put into the culture dish after sterilization. MG63 cells with a concentration of 7×10^4^ cells/cm^2^ were mixed thoroughly and then added to the culture dish. The plates were incubated at a 5% CO_2_, 37°C and 100% humidity incubator. Cells were cultured 24 hours later to replace the osteogenic differentiation medium. Alkaline phosphatases (ALP) activity was determined on the 7th and 14th day of osteoinduction. ALP levels were normalized to the total protein content at the end of the experiment. The expression levels of osteogenic-related genes, RUNX2 and OCN were detected on 21 Days of osteoinduction. The image adoption was performed using gel doc XR system (Bio-Rad, USA).

### Statistics

Each in vitro experiment was repeated at least three times, data were statistically analyzed by a t-test analysis using “GraphPad Prism” software. Significant differences were determined for p<0.05, and p<0.01 was considered highly significant.

## Results

### Surface morphology

**Fig 1:**
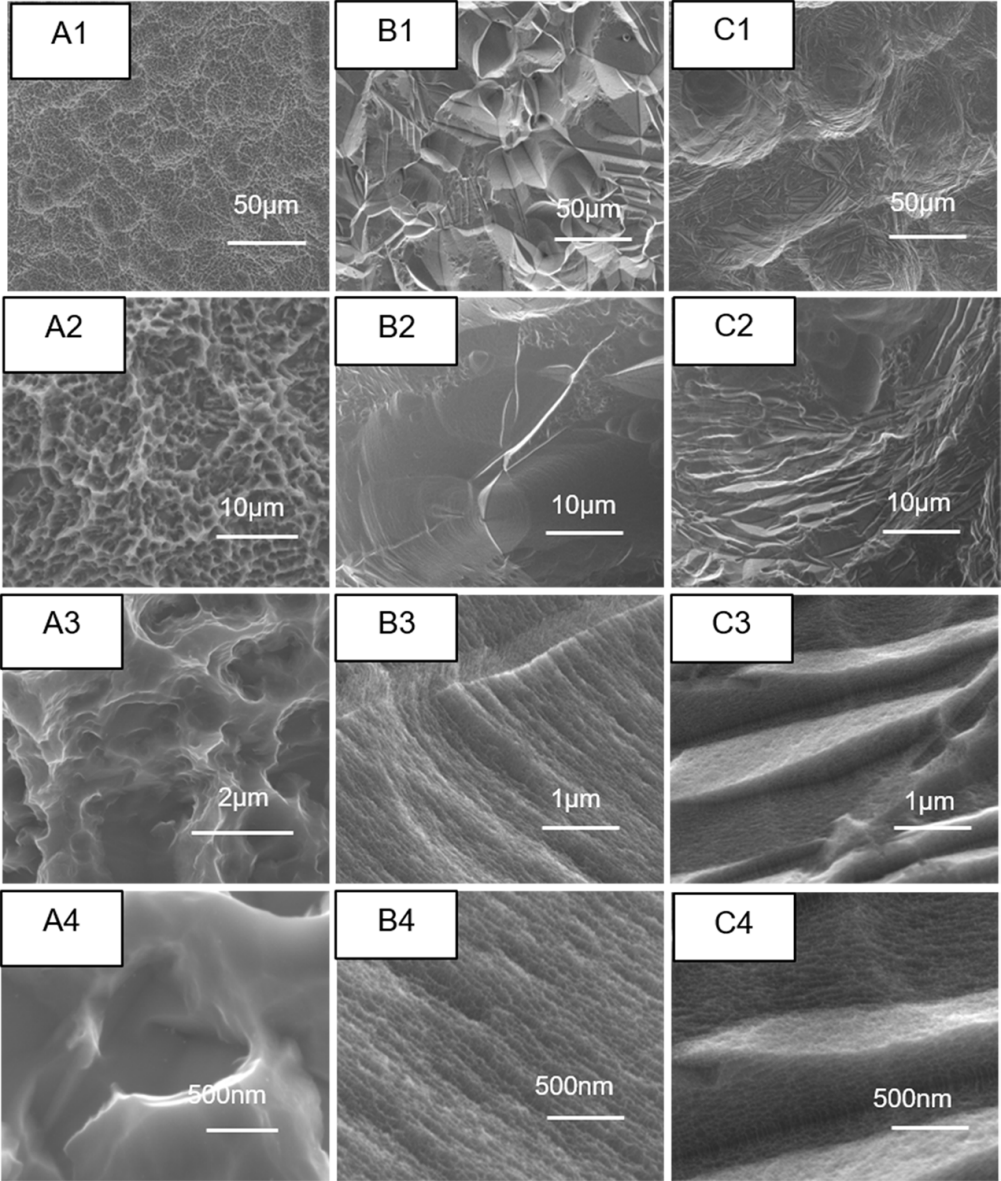
Surface morphology of SLA(A), E(B), SE(C) samples.

Fig 1 presents the morphologies of SLA (A), E (B) and SE (C) surfaces. It can be seen in Fig 1A that SLA shows a continuous honeycomb-like morphology with holes of ~2 μm. Besides, the surface of SLA is a micron-submicron roughened but smooth at the nanoscale (Fig 1A4), which is free of nanostructures.

Fig 1B shows the morphology of the E surface after etching with a mixture of NH_4_OH and H_2_O_2_. It can be seen that a large number of holes with diameters of 10~30μm evenly appears on the porous surface (Fig 1B1). The walls of these holes are covered with a lamellar fringe pattern (Fig 1B2 and 1B3), and these fluctuant textures are also densely distributed with tens of nanometers pits (Fig 1B4) and the distribution is relatively uniform. In addition, the sandblasting pretreatment process was used before the etching of concentrated H_2_O_2_ and NH_4_OH mixture in order to further coarsen the surface, and the SE surface as shown in Fig 1C were obtained. The morphologies of SE samples show a spherical sandblasting hole structure at micro level (Fig 1C1) with the inner walls which are combined with a few micrometer-sized microgrid (Fig 1C2 and 1C3), and the grooves surface is also filled with a dense nano pits (Fig 1C4). Moreover, the inner walls of the SE surface are rougher than those of the E surface, and its nanostructures are not plainly distributed but along with the distribution of micro grooves structure fluctuation. Therefore, it can be considered that the SE surface has a multi-stage roughed structure with micron/sub-micron/nanometer structures.

### Surface chemistry

**Fig 2:**
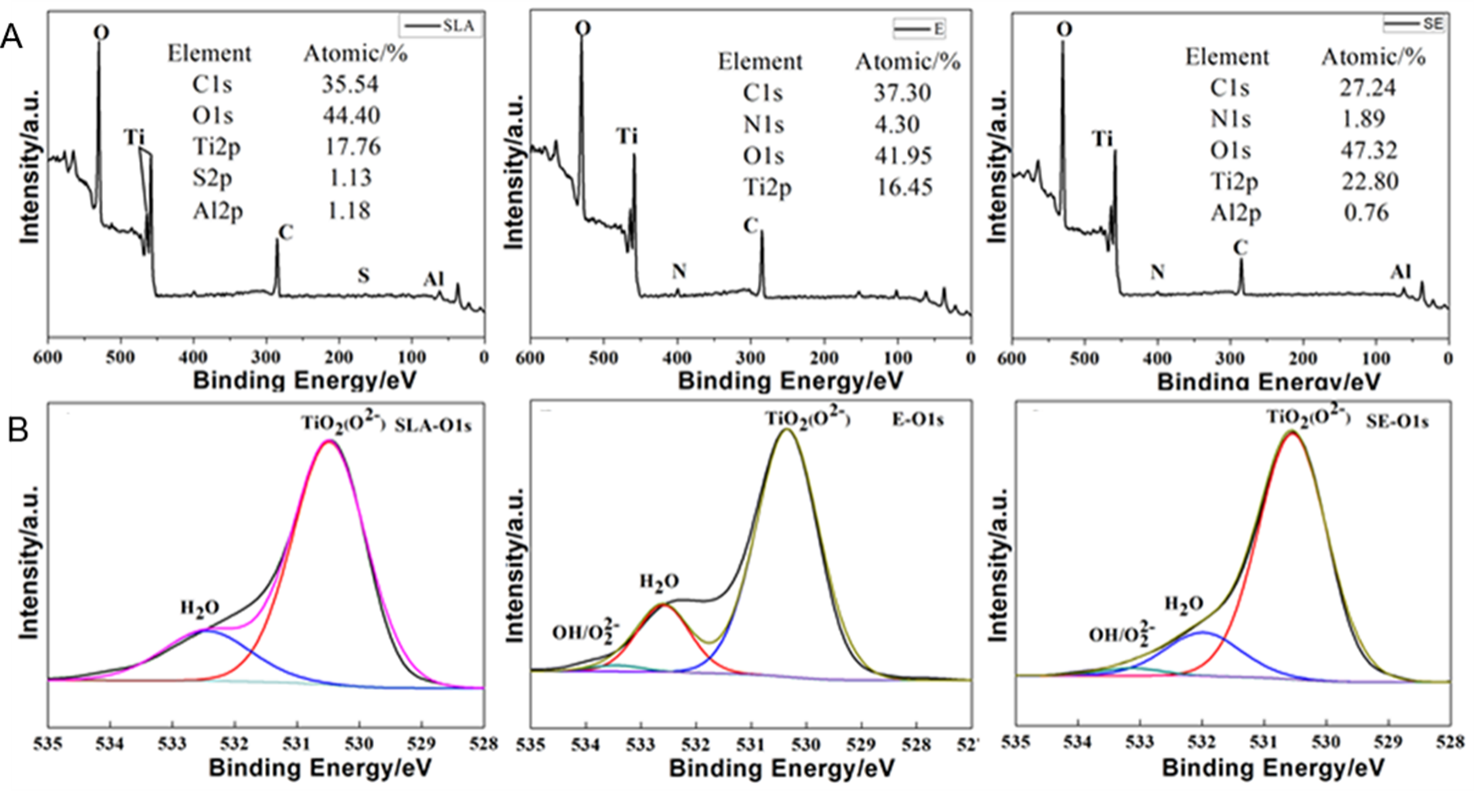
XPS survey date of SLA, E, SE surface(A) and XPS narrow spectra of O1s on SLA, E, SE surface(B).

The XPS survey spectra of SLA, E, and SE titanium surfaces are depicted in Fig 2A. It can be observed that all the titanium surfaces are mainly composed of O, Ti and C elements, and there is a very small amount of N element on the E and SE surfaces, which may be due to the reactions of NH_4_OH with titanium or from the atmosphere.

According to the O1s spectra obtained from the samples (Fig 2B), the main chemical species on the surfaces were postulated to be O^2-^, H_2_O corresponding to the peaks centered at 530.3 eV, 532.8 eV, respectively, but there are some differences in their intensities. In addition, it can be seen from the O1s spectra results of E and SE that another peak centered at 533.5 eV may also be O_2_^2-^ group according to NIST XPS Database for E and SE titanium surfaces. Due to the formation of Ti(H_2_O_2_)_2_^4+^ during the etching process, a small amount of O_2_^2-^ residue may also be detected by XPS.

### Surface wettability and roughness

**Fig 3:**
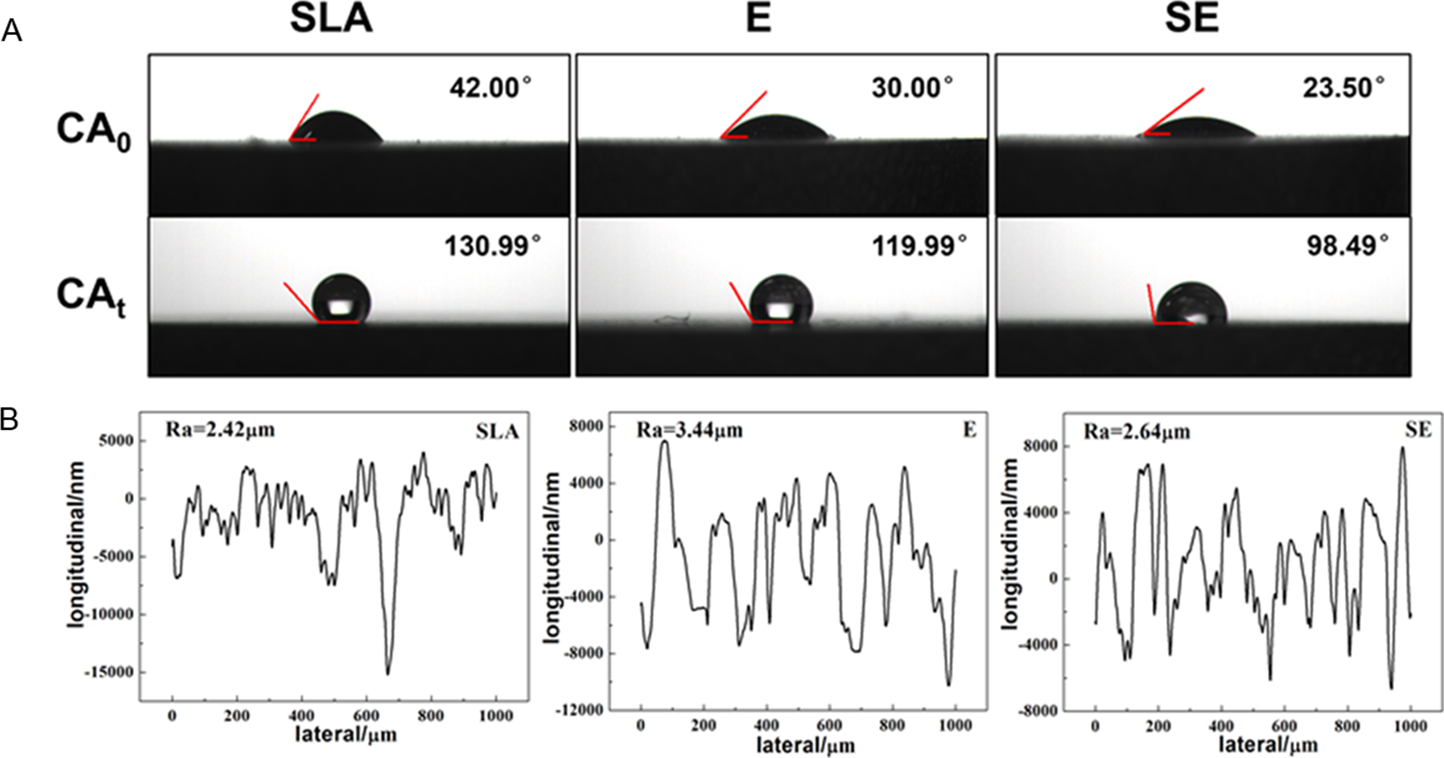
Water contact angle on SLA, E, SE surface(A), CA_0_: fresh surface, CA_t_: exploded surface and Surface profiler test results of SLA, E, SE surface(B).

Both surface wettability and roughness are the important properties to reveal surface characteristics. The surface contact angles of fresh surfaces and exploded surfaces were shown in Fig 3A. It can be seen that the contact angles of the fresh surfaces of E and SE are smaller than that of SLA, indicating that E and SE samples possess better hydrophilicity than SLA sample. When exposed to the air for a period, the contact angles of three surfaces are all greater than 90°. This may dues to the contamination of titanium surfaces. However, the contact angle of SLA is still the largest. In contrast, the change of the contact angle of SE is the smallest, indicating that its retention ability of hydrophilicity is better.

Fig 3B shows the surface profiler test results of SLA, E, and SE samples. The surface roughness of SLA, E, SE is 2.42 μm, 3.44 μm, and 2.64 μm, respectively. The hole depth of E and SE surfaces is 8–15 μm, while that of SLA surface is about 7 μm, and the roughness of the former etching surface is greater than that of SLA. In general, a similar surface roughness to SLA can be obtained by the mixture of NH_4_OH and H_2_O_2_. In addition, the surface roughness of SE sample is also the same as that of SLA, but lowers than that of E. And from Fig 3B we can see that SE surface has a more intensive fluctuation than E surface. Combined with SEM morphologies of Fig 1, the SE surface is a more complex rough structure compared to the E surface.

### Cell analysis

**Fig 4:**
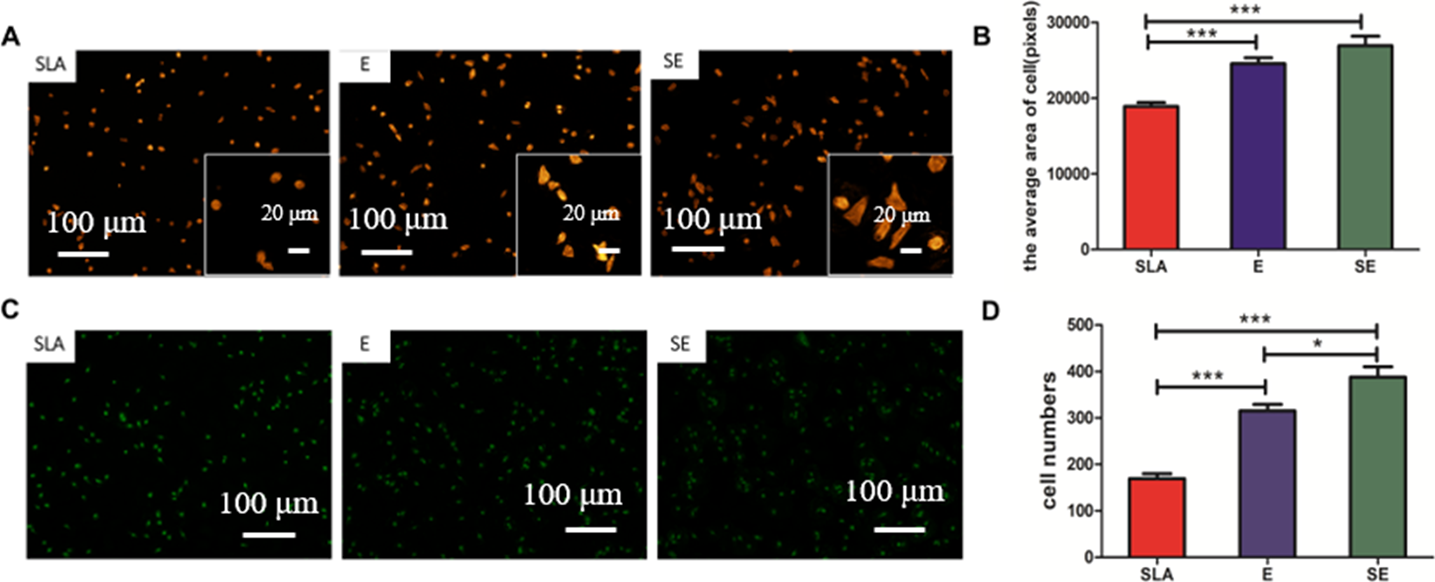
The average area and number of MG63 cells attached to SLA, E, SE surface after cultured for 4 hours. A: Cytoskeleton rhodamine-phalloidin staining chart, the lower right corner for its enlarged image; B: The individual cell spreading area on the surface (*** P <0.001); C: Cell nucleus acridine orange(AO) staining; D: Cell counting(*P<0.05;***P<0.001).

Fig 4 shows the average area and numbers of MG63 cells attached to SLA, E, SE surfaces after culturing for 4 hours. Fluorescence microscopies show that the number and morphologies of MG63 cells are different after 4 hours of inoculation. In Fig 4A, cells on the SLA surface are rounded, while those on the E and SE surfaces are mostly spindle or polygonal. In combination with Fig 4B, the spreading area of cells on SLA surface is also smaller than that of E and SE surfaces. And the difference between them is highly significant (P < 0.001), indicating that the cells on E and SE surfaces grow better. The area of cells on E surface is larger than that of SE, but there is no significant difference between them (P > 0.05). The results of Fig 4C and 4D show that the numbers of adherent cells on E and SE surfaces are both higher than that of SLA, and the difference is particularly significant (P < 0.001). Even more, the number of adherent cells on SE surface is slightly higher than that of E, and there is a statistically significant difference between them (P < 0.05).

**Fig 5:**
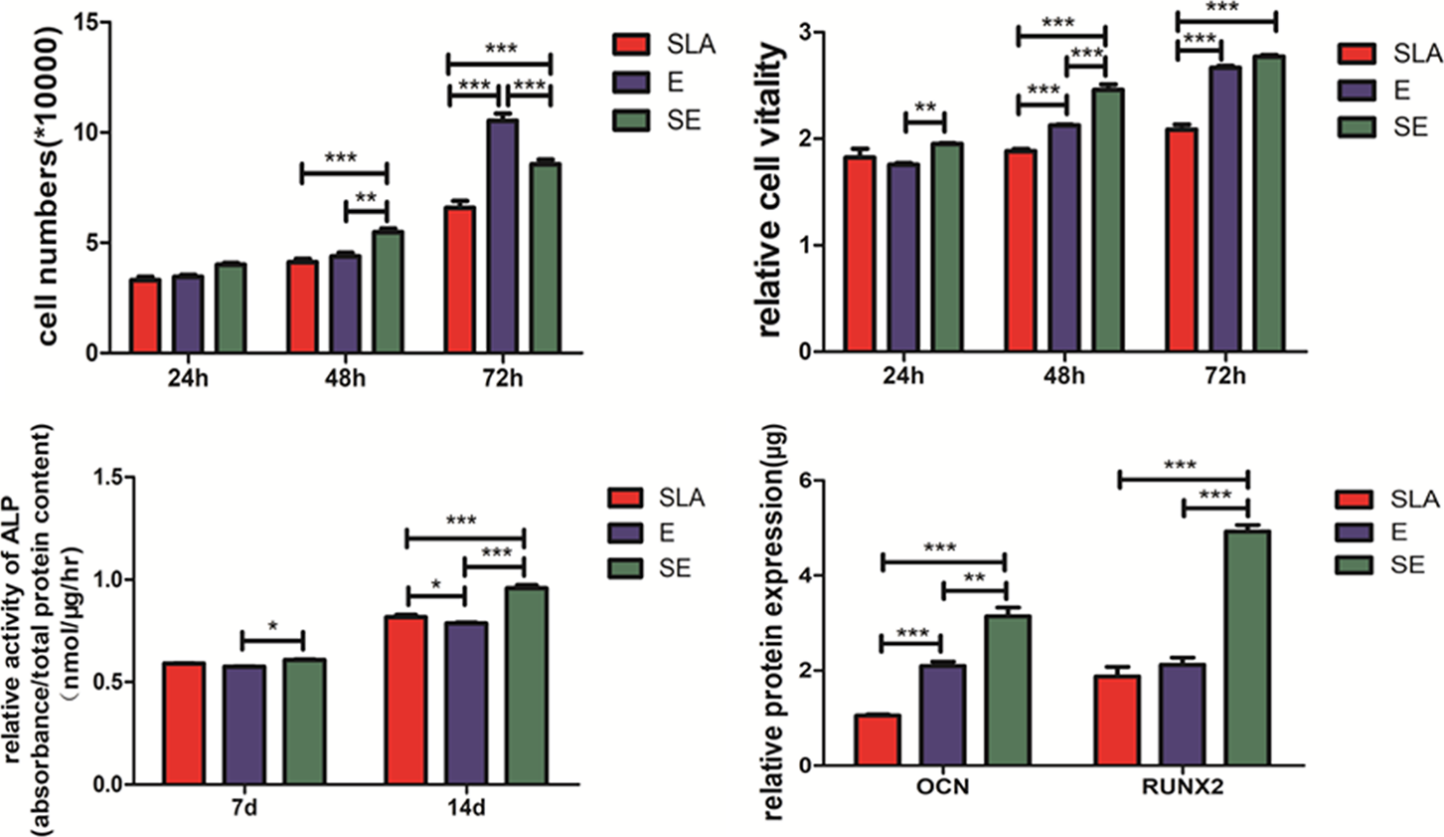
Cell analysis on SLA, E, SE surface. A: Cell numbers after cultured for 24, 48 and 72 h; B: Cell viability after cultured for 24, 48 and 72 h; C: Relative activity of ALP after 7 d and 14 d of osteoinduction; D: OCN and RUNX2 production after 21-day osteoinduction(*P<0.05;**P<0.01;***P<0.001).

Fig 5A shows the cell proliferation of three surfaces at 24 h, 48 h and 72 h. There are no significant differences at 24 h (P > 0.05). At 48 h, the number of cells increases slightly, and SE surface is the highest and it is significantly different from E and SLA (P< 0.01). There is no significant difference between E and SLA. When the time comes to 72 h, it depicts that E and SE surfaces have higher cell number than SLA (p < 0.001) and faster rate of increment. At the same time, E surface has the highest number of cell proliferation, playing a strong role in promoting proliferation. Overall, titanium plates treated with a mixture of H_2_O_2_/NH_4_OH have a better effect on the proliferation of MG63 cells than SLA treatment. Additionally, the SE is more stable in promoting cell proliferation.

Cell vitality was measured by cck-8 assay. It can be seen from Fig 5B that the cell activity of SE surface is the highest at 24 h, and there is significant difference with E samples (p < 0.01). But there is no significant difference between SLA and E/SE. At 48 h, the cell viability of SE is the highest, and the difference with E surface is also significant (p < 0.001). However, there is no significant difference between SE and E (p > 0.05) at 72 h, but much higher than that of SLA. It indicates that the etching of H_2_O_2_/NH_4_OH has a more positive effect on the cell activity of implant surfaces than that of SLA.

Fig 5C shows the results of relative activity of ALP after 7 d and 14 d of osteoinduction. SE surface has always maintained the highest ALP activity. At 14 days, the ALP activity of E surface is slightly lower than the SLA, and there is a statistically significant difference between the two (p < 0.05). It can be seen that the surface treated with H_2_O_2_/NH_4_OH can promote the osteogenic differentiation of MG63 cells well.

Fig 5D shows the results of OCN and RUNX2 production after 21-day osteoinduction. The results show that cells cultured on H_2_O_2_/NH_4_OH treated surfaces produced the most RUNX2 and OCN, and especially SE possesses significantly higher RUNX2 and OCN content than other group (P < 0.001), but there is no significant difference between SLA and E (p > 0.05) for RUNX2 protein expression.

## Discussion

In this study, two novel pure titanium surfaces (E and SE surfaces) were fabricated by using H_2_O_2_/NH_4_OH mixed solution, which is a simple, cheap and eco-friendly method to obtain micro/nano structure surface. The reaction of a mixture of NH_4_OH and H_2_O_2_ with the titanium sheet is mainly due to the coordination between them and the oxidation of H_2_O_2_. NH_4_OH. H_2_O_2_ is a mixture of weak base and weak acids, the etching occurs mainly at the grain boundaries enriched with impurity elements without H_2_. It is clear that the micro/nano hierarchical structures have been obtained in titanium surfaces after etching in a mixture of NH_4_OH and H_2_O_2_. The micro holes of 10~30 μm on the surface are very close to the size of osteoblasts, which can be used as good cell implantation points. On the other hand, the density of surface holes can meet the requirements, while the distribution of gully morphology can also promote the extension of cell pseudopodia, all of which hints that good biological effects will be obtained. Furthermore, the lamellar fringe morphologies, especially in the SE surface, not only significantly increase the adhesion area of the cells, but also appear to be similar to the human bone structure, which seems to be an imitated bone structure. Studies have shown that a similar micro-groove morphology can also promote the contact guidance, a phenomenon where cells align themselves and migrate along the grooves [30–32].It is believed that the contact guidance on cell proliferation, adhesion, and gene expression have a very positive impact.

According to the XPS results, a small amount of O_2_^2-^ vestigial also is detected. O_2_^2-^ not only keep the titanium surface clean, but also significantly improve the antimicrobial properties, promote wound healing and ameliorate the initial stability of the implant [33, 34]. The existence of these reactive oxygen species on the surface can destroy the bacterial cell membrane, inhibit bacterial growth, and raise the activity of adherent cells [28, 29, 35]. In general, compared with SLA surface, E and SE surfaces have higher oxygen contents. They bring continuous oxygen supply can enhance the surface wettability and further improve the initial chemical environments of the implant and promote osteoblast adhesion [36]. It means that the surface modification method of E and SE will effectively ameliorate the osseointegration performance of the implant.

For the fact that organic ions and a large amount of organic matter exist in the form of aqueous solutions in vivo, the combination of water and implant surface plays an important role in a cascade of biological behaviors, such as the cell and protein adhesion. As can be seen from Fig 3A, the E and SE surfaces both have superior hydrophilicity to SLA and more retention of wettability. The reason may be that these two kinds of surfaces with densely porous structures are more susceptible to the moisture absorption and the composite nanostructures on their walls enhance the capillary effect, prohibiting the desorption of the water [37, 38]. On the other hand, it may be related to the chemical compositions of the etching surfaces, such as the presence of hydrophilic ROS on the surfaces and the formation of Ti(H_2_O_2_)_2_^4+^ which may decompose to form hydrophilic functional groups.

**Fig 6:**
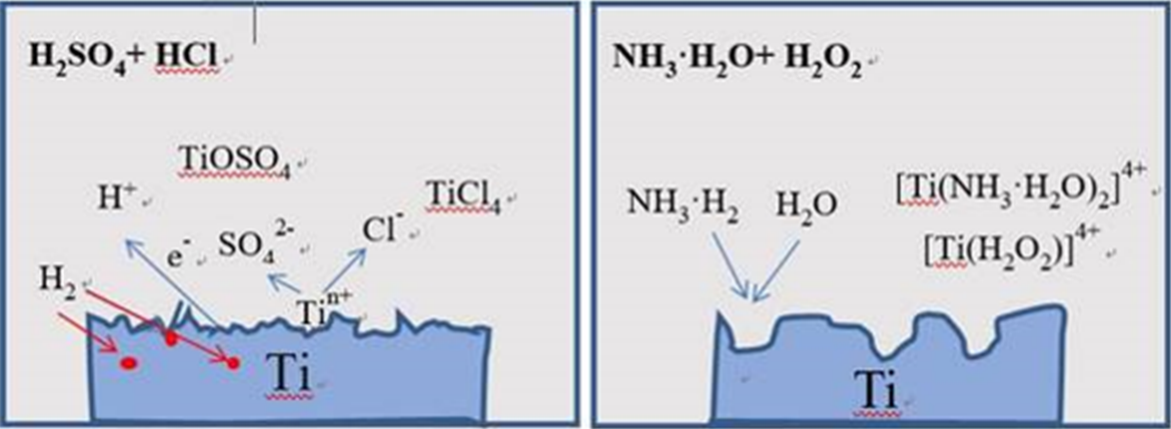
The corrosion mechanisms of H_2_SO_4_ + HCl and NH_4_OH + H_2_O_2_.

The corrosion mechanisms of different methods need to be considered as well and they are schematically described in Fig 6[39]. Micro holes were formed in E and SLA. NH_4_OH and H_2_O_2_ with strong coordinative capability etched titanium at the boundaries very fast to form very huge and deep holes. H_2_SO_4_ and HCl can form many complex compounds with titanium and impurities and also directly etch titanium both in boundaries and facet. It leads different micron- scale surface. The SLA surface has peak valley morphology which is easy to bring the defects, while E surface has hole pit morphology with larger curvature as well as relatively smooth and flat hole wall.

$$H_{2}SO_{4}+Ti\to TIOSO_{4}+H_{2}$$ (1)

$$\text{HCl}+\text{Ti}\to TiCl_{4}+H_{2}$$ (2)

$$NH_{3}.H_{2}O+Ti\to {\left[Ti{\left(NH_{3}.H_{2}O\right)}_{2}\right]}^{4+}$$ (3)

$$H_{2}O_{2}+Ti\to {\left[Ti\left(H_{2}O_{2}\right)\right]}^{4+}$$ (4)

Besides, a similar surface roughness to SLA can be obtained by the mixture of NH_4_OH and H_2_O_2_. The titanium plate etched with a mixture of NH_4_OH and H_2_O_2_ is clean without any toxic elements, which also helps to improve the hydrophilicity and biocompatibility of the material. Together with simple process and lower cost, it is an ideal method for the preparation of surface micro/nano hierarchical structures.

The results of the above biological experiments reveal that titanium plates etched with a mixture of NH_4_OH and H_2_O_2_ can significantly promote cell adhesion, proliferation and differentiation compared to the SLA surface. The porous surface obtained by H_2_O_2_/NH_4_OH has an ideal surface roughness as well as significantly increasing the surface area of the implant. While the hole walls combined with the nano trenches and nano pits provide more spaces and mechanical locking opportunities for cell adhesion. Meanwhile, a larger contact area between the cells and the material also promote the extension of cell pseudopodia and improve the bonding strength of tissue-bone interface. Therefore, it is more conducive to cell attachment and colonization, and easier to induce and promote osseointegration.

**Fig 7:**
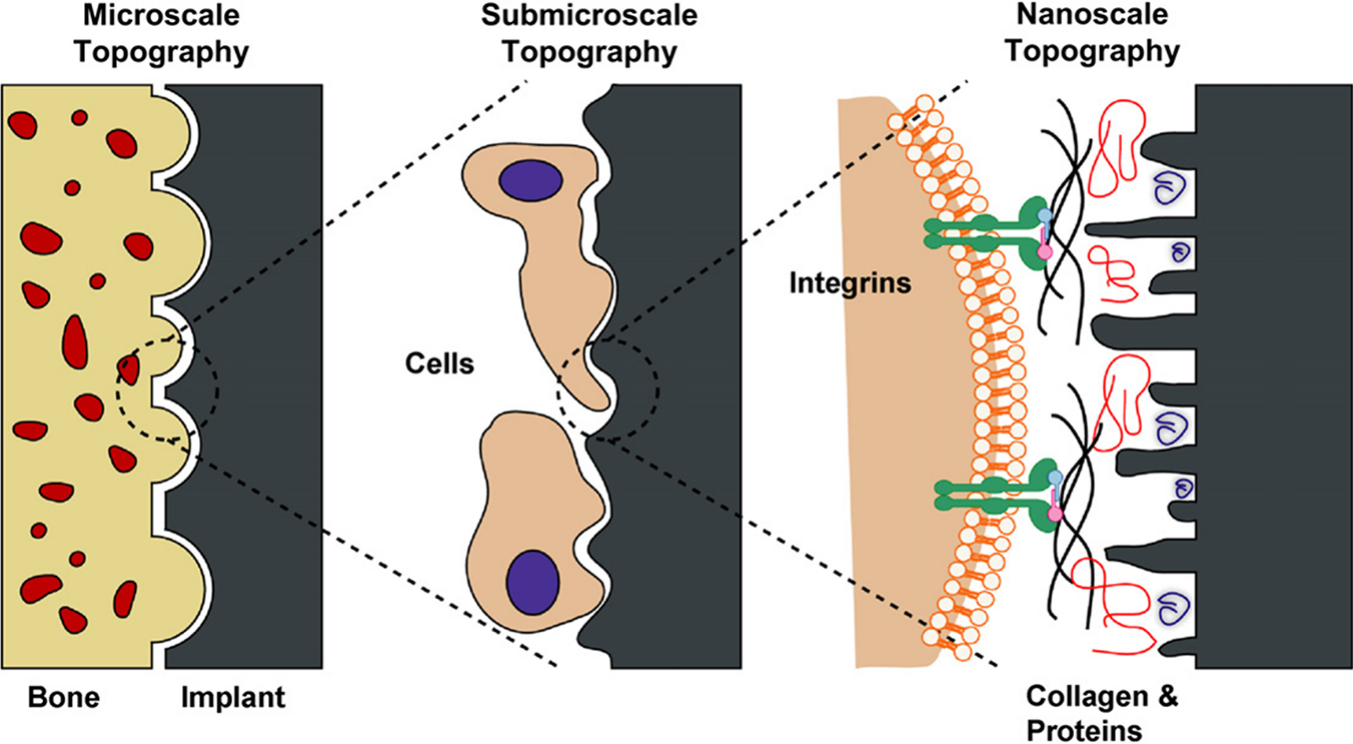
Schematic of the interactions between bone and the implant surface at different topographical scales.

Several studies have shown that the effect of surface structure on cell response and some mechanism have been reported and researched, as well as cell toxicity [40–42]. Verma S K et al. also focus on the relation of TiO2 nanoparticles and cell cycle, including cell toxicity [43–44]. Rolando et al. [45] have observed that the osteoblast increased bone-to-implant contact has been enhanced with the increasement of micro- and submicro- surface structures and the proper feature sizes suitable for cell dimensions (Fig 7). And the nanotopography plays an important role in osteoblast differentiation and tissue regeneration differentiation and local factor production in vitro. In this study, the SE specimens possess micro-, submicro- and nano- surface structures simultaneously, leading to the better cell adhesion. In addition, the hierarchical surface structures of SE provide the increased surface area and high wettability and surface free energy, which enhanced the cell adhesion. Moreover, the increased nano-scale structures are able to improve cell response. Because cells have nano structure surface and they live inside an extracellular matrix (ECM) with nano-scale collagen fibrils [46]. Fluorescence microscopies results (Fig 4) indicate that the E and SE surfaces are mostly spindle or polygonal with improved pre-osteoblasts adhesion, showing better spreading of pre-osteoblasts. Another possible explanation hypothesis for the better bone regeneration of nanotopography could be that nanotopographical features modulate mechanotransduction cell pathways with the mechanical forces transmitted and guide the process of bone regeneration [47]. And this hypothesis need further test in the future study.

Natural bone tissue has a composite structure containing micrometer and nanoscale structure. The micro/nano hierarchical structures can be constructed on implant surfaces, which can better simulate the structure of natural bone tissue and achieve the purpose of bone fusion. Many researchers claimed that micro/nano hierarchical structure could significantly improve cell adhesion, proliferation and differentiation [14, 48,49], which is consistent with the results obtained in this paper. Compared with the micro-morphologies of SLA samples, micro/nano hierarchical morphologies are superior to improve ALP activity and promote gene expression. Isa et al. [50] have found that the expression of RUNX2, a transcription factor that plays a key role in osteoblast differentiation, only increases on the surface with nanostructures. Guo et al. [51] demonstrated that nano-structured surfaces would benefit the increase of bone sialoprotein, osteopontin, osteocalcin, etc. On the whole, the surface morphologies etched by a mixture of NH_4_OH and H_2_O_2_ can enhance the cells biological response well, which is beneficial in the design of dental implants.

## Conclusions

The effect of titanium implant surface morphology on its biological behavior has been the important point of the study on the surface modification of implants. In this paper, the titanium surfaces were modified by a mixture of NH_4_OH and H_2_O_2_, and the micro/nano hierarchical structure was successfully fabricated on the surface of titanium plate. These surfaces possess ROS and higher oxygen concentration as well as similar roughness and superior hydrophilicity to SLA samples. The micron holes of 10~30 μm on the surface are very close to the size of osteoblasts and additional nanostructures provide a bionic environment for osteocytes. Cell experiments also demonstrated that MG63 cells cultured on these two-novel micro/nano structured titanium surfaces exhibited enhancing adhesion, proliferation, vitality and osteogenic differentiation, especially SE samples. What’s more, etching with H_2_O_2_/NH_4_OH mixed solution is a simple, cheap and ecofriendly surface treatment method. This may be a promising treatment to titanium implant.
